# Monoclonal Antibodies in the Treatment of Relapsing Multiple Sclerosis: an Overview with Emphasis on Pregnancy, Vaccination, and Risk Management

**DOI:** 10.1007/s13311-022-01224-9

**Published:** 2022-04-04

**Authors:** Nik Krajnc, Gabriel Bsteh, Thomas Berger, Jan Mares, Hans-Peter Hartung

**Affiliations:** 1 Department of Neurology, Medical University of Vienna, Vienna, Austria; 2 Department of Neurology, Palacky University Olomouc, Olomouc, Czech Republic; 3 Department of Neurology, Medical Faculty, Heinrich-Heine University, Moorenstrasse 5, 40225 Düsseldorf, Germany; 4 Brain and Mind Center, University of Sydney, Sydney, Australia

**Keywords:** Multiple sclerosis, Disease-modifying therapy, Monoclonal antibodies, Natalizumab, Alemtuzumab, Rituximab, Ocrelizumab, Ofatumumab, Ublituximab

## Abstract

Monoclonal antibodies have become a mainstay in the treatment of patients with relapsing multiple sclerosis (RMS) and provide some benefit to patients with primary progressive MS. They are highly precise by specifically targeting molecules displayed on cells involved in distinct immune mechanisms of MS pathophysiology. They not only differ in the target antigen they recognize but also by the mode of action that generates their therapeutic effect. Natalizumab, an $\alpha$_4_$\beta$_1_ integrin antagonist, works via binding to cell surface receptors, blocking the interaction with their ligands and, in that way, preventing the migration of leukocytes across the blood–brain barrier. On the other hand, the anti-CD52 monoclonal antibody alemtuzumab and the anti-CD20 monoclonal antibodies rituximab, ocrelizumab, ofatumumab, and ublituximab work via eliminating selected pathogenic cell populations. However, potential adverse effects may be serious and can necessitate treatment discontinuation. Most importantly, those are the risk for (opportunistic) infections, but also secondary autoimmune diseases or malignancies. Monoclonal antibodies also carry the risk of infusion/injection-related reactions, primarily in early phases of treatment. By careful patient selection and monitoring during therapy, the occurrence of these potentially serious adverse effects can be minimized. Monoclonal antibodies are characterized by a relatively long pharmacologic half-life and pharmacodynamic effects, which provides advantages such as permitting infrequent dosing, but also creates disadvantages regarding vaccination and family planning. This review presents an overview of currently available monoclonal antibodies for the treatment of RMS, including their mechanism of action, efficacy and safety profile. Furthermore, we provide practical recommendations for risk management, vaccination, and family planning.

**Supplementary Information:**

The online version contains supplementary material available at 10.1007/s13311-022-01224-9.

## Introduction

Multiple sclerosis (MS) is an autoimmune inflammatory disease of the central nervous system (CNS) that affects about 2.8 million people worldwide [1]. The majority of patients (85%) initially follow a relapsing course (RMS), defined by acute exacerbations and periods of relative clinical stability in between [2]. Disability is accrued associated with relapses but occurs also independent of them. Over the last quarter of a century, an ever-increasing number of disease-modifying treatments (DMTs) has emerged, enabling effective reduction of disease activity, i.e., occurrence of relapses and T2-hyperintense lesions (T2L) contrast-enhancing lesions (CEL) on MRI, and to a lesser degree also disability progression [3]. As the disease course displays a considerable degree of both inter- and intra-individual variation, treatment choices depend on assessing the disease stage and judging the current level of disease activity. Across differing definitions, RMS may be classified as highly active based on the number and severity of relapses in the past 1–2 years, the number of new and/or enlarging T2L and/or Gd-enhancing lesions on MRI, or an insufficient response to treatment with one or more disease-modifying therapy (DMT) for at least one year [4]. Highly active RMS requires highly effective DMT (HET), which is almost exclusively achieved by monoclonal antibodies (mAb).

The advent of mAb has revolutionized treatment of MS due to their targeted mechanism, potent efficacy and favorable risk profile. They were originally developed from mice to prevent organ rejection in 1986; however, reactions to murine mAbs were soon associated with antidrug antibodies which led to the development of chimeric mouse-human mAbs [5, 6]. To minimize risks, particularly the risk of allergic or infusion-related reactions (IRRs), mAb have undergone several engineering generations to humanize their components in the last decades. This renders them less immunogenic and less likely to evoke generation of anti-drug antibodies. Also this increases clearance times [7]. The first-generation biologics were entirely murine in structure, sometimes leading to potentially fatal immune responses. Second-generation biologics were engineered as either chimeric (combining human Fc-regions with murine variable regions) or humanized (the variable region containing relatively more human protein). Third generation biologics are fully human mAb, yet these still appear to induce production of anti-human mAb. The mAb currently licensed for in MS have proven high efficacy in phase 3 studies and are therefore used in patients with high disease activity. Labels given by regulatory agencies in different countries vary. While all mAbs are approved to treat relapsing forms of MS in the USA, none of those are licensed in Europe for use in less active disease, based on weighing benefits vs. risks.

**Fig. 1:**
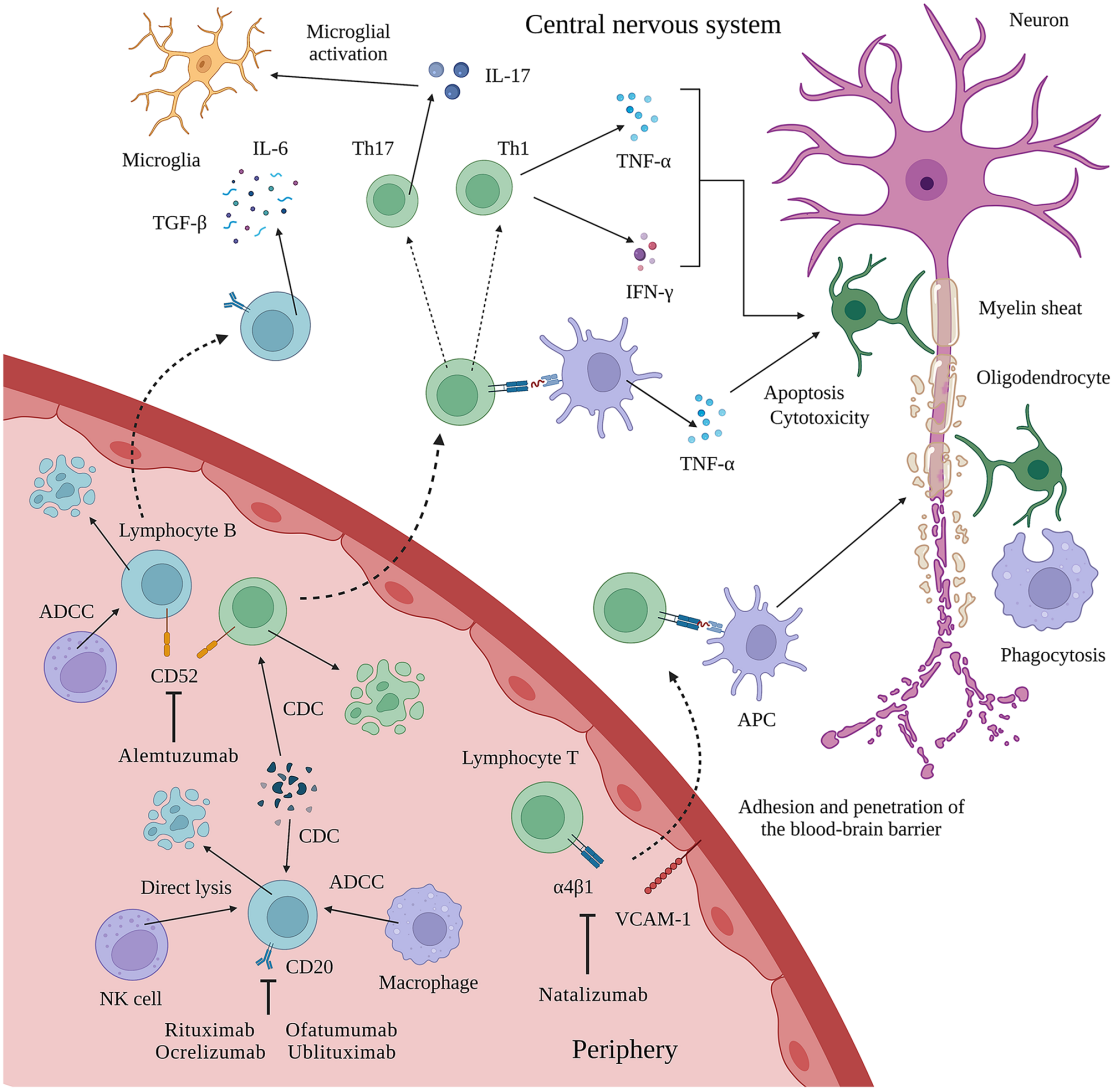
Mechanism of action of mAb in the treatment of multiple sclerosis. Rituximab, ocrelizumab, ofatumumab, and ublituximab target CD20 expressing lymphocytes B causing ADCC and CDC of circulating lymphocytes B. Alemtuzumab targets CD52 expressing lymphocytes, eosinophils, monocytes/macrophages, and dendritic cells, resulting in their rapid depletion. Natalizumab binds to $\alpha$4$\beta$1 integrin receptor on endothelial cells, preventing interaction between $\alpha$4$\beta$1 integrin and VCAM-1 and, therefore, inhibiting migration of leukocytes through the BBB into the CNS parenchyma. Created with BioRender.com. ADCC antibody-dependent cell-mediated cytolysis, APC antigen-presenting cell, CD cluster of differentiation, CDC complement-dependent cytolysis, IL interleukin, TGF-$\beta$ transforming growth factor $\beta$, TNF-$\alpha$ tumor necrosis factor $\alpha$, VCAM-1 vascular cell adhesion molecule 1

mAbs belong to the immunoglobulin G (IgG) isotype which bind specifically with their fragment antigen-binding (Fab) region to the epitope of the target molecule. The latter can either inhibit a specific function or directly induce an intracellular signaling. The binding of the fragment-crystallizable (Fc) region can lyse a target cell through either antibody-dependent cell-mediated (ADCC) or complement-dependent cytotoxicity (CDC) [8]. However, they differ not only in the target antigen they recognize but also regarding the mechanism by which they exert their therapeutic effect (Fig. 1). Natalizumab, for instance, works via binding to cell surface receptors, blocking interaction with their ligands and, thereby prevents the transition of leukocytes across the blood–brain barrier (BBB). On the other hand, alemtuzumab and the class of anti-CD20 mABs rituximab, ocrelizumab, ofatumumab, and ublituximab work via killing selected cell populations. Potential adverse effects may be serious and can necessitate treatment discontinuation. Such serious adverse events are the risk for (opportunistic) infections, autoimmune diseases or malignancies.

mAb are characterized by a relatively long pharmacologic half-life (IgG subclasses up to 30 days) and pharmacodynamic effects [9, 10], which provide advantages such as permitting infrequent dosing, but also create disadvantages regarding vaccination and family planning.

Here, we provide an overview of mAb for RMS treatment with a special focus on potential side effects and risk management, pregnancy and family planning, and vaccinations (Tables 1 and 2).

**Table 1:** mAbs, their characteristics, important safety issues, and proposed management of potential risks

**DMT**	**Mechanism of action**	**Dose, administration and interval of application**	**Efficacy**	**Important safety issues**	**Risk management**
Natalizumab	$\alpha$_4_$\beta$_1_ integrin antagonist	Dose: 300 mg iv. or sc Every 4 weeks (SID) to every 6 weeks (EID)	Phase 3 clinical trials: AFFIRM, SENTINEL Clinical outcomes • 24–42% decrease in the risk of sustained disability progression • 54–68% reduced ARR at year 1 MRI outcomes • 83% and 89–92% reduction of the number of new and/or enlarging T2L, and Gd-enhancing lesions at year 2, respectively	PML	Monitoring anti-JCV antibody index, MRI monitoring
Alemtuzumab	Anti-CD52 mAb	Dose: 12 mg iv First cycle: 5 consecutive days Second and/or further cycles (≥ 12 months from the last cycle): 3 consecutive days	Phase 3 clinical trials: CARE-MS I, CARE-MS II Clinical outcomes • 49.4–54.9% reduced ARR • 65–78% of patients relapse-free at year 2 MRI outcomes • 46% patients with new or enlarging T2L (68% on IFN $\beta$-1a) • 9% patients with Gd-enhancing lesions at year 2 (23% on IFN $\beta$-1a) • 25–40% decreased rate of brain atrophy	Infections, hypo- and hyperthyroidism, immune thrombocytopenic purpura, nephropathy, acquired hemophilia A, autoimmune hepatitis, hemophagocytic lymphohistiocytosis, IRR, hemorrhagic stroke, myocardial ischemia, pericarditis, pulmonary alveolar hemorrhage	Before treatment: • Screening for chronic infections^a^ • Vaccination status^b^ • Listeria-free diet^c^ During treatment: • Premedication (methylprednisolone, antihistaminics, antipyretics) • ECG before application • Heart rate and blood pressure monitoring during application • Laboratory tests^d^ • Platelet counts on 3rd and 5th day of application • Prevention of infection/reactivation with herpes virus^e^ and listeria^c^
Rituximab	Anti-CD20 mAb depleting lymphocytes B	Dose: 500–1000 mg iv Every 6–12 months (some protocols initiate the treatment with two applications 2 weeks apart)	No phase 3 clinical trials	Infections, IRR, hepatitis B reactivation, hypogammaglobulinemia	Before treatment: • Screening for chronic infections^a^ • Vaccination status^b^ During treatment: • Premedication (methylprednisolone, antihistaminisc, antipyretics) • Prophylaxis before hepatitis B reactivation at carrier-patients, immunoglobulin level monitoring, screening for malignancies
Ocrelizumab	Anti-CD20 mAb depleting lymphocytes B	Dose: 600 mg iv Every 6 months (apart from the first two cycles with 300 mg two weeks apart)	Phase 3 clinical trials: OPERA I, OPERA II Clinical outcomes • 40% decrease in the risk of sustained disability progression • 46–47% reduction of ARR MRI outcomes • 94–95% lower number of Gd-enhancing lesions	Infections, malignancies (breast cancer), IRR, hepatitis B reactivation, hypogammaglobulinemia	Before treatment: • Screening for chronic infections^a^ • vaccination status^b^ During treatment: • Premedication (methylprednisolone, antihistaminics, antipyretics) • Prophylaxis before hepatitis B reactivation at carrier-patients • Immunoglobulin level monitoring • Screening for malignancies
Ofatumumab	Anti-CD20 mAb depleting lymphocytes B	Dose: 20 mg sc Every 28 days (apart from first applications on 1st, 8th, and 15th day)	Phase 3 clinical trials: ASCLEPIOS I, ASCLEPIOS II Clinical outcomes • 32–34% decrease in the risk of sustained disability progression • 50–60% reduction of ARR MRI outcomes • 82–85% lower number of new or enlarging T2L • 94–97% lower number of Gd-enhancing lesions	Infections, injection-related reactions, hepatitis B reactivation, hypogammaglobulinemia	Before treatment: • Screening for chronic infections^a^ • Vaccination status^b^ During treatment: • Prophylaxis before hepatitis B reactivation at carrier-patients • Immunoglobulin level monitoring • Screening for malignancies
Ublituximab	Anti-CD20 mAb depleting lymphocytes B	Dose: 450 mg iv Every 24 weeks (apart from 150 mg on day 1 and 450 mg on day 15)	Phase 3 clinical trials: ULTIMATE I, ULTIMATE II Clinical outcomes • 49.1–59.4% reduction in ARR • 34.3% reduction in 24-week confirmed disability progression MRI outcomes • 90.0–92.4% lower number of new or enlarging T2L • 96.5–96.7% lower number of Gd-enhancing lesions	IRR, infections, hepatitis B reactivation, hypogammaglobulinemia	Before treatment: • Screening for chronic infections^a^ • Vaccination status^b^ During treatment: • Prophylaxis before hepatitis B reactivation at carrier-patients • Immunoglobulin level monitoring • Screening for malignancies

*ARR* annualized relapse rate, *DMT* disease-modifying therapy, *HIV* human immunodeficiency virus, *IRR* infusion-related reaction, *JCV* John Cunningham virus, *mAb* monoclonal antibodies, *PML* progressive multifocal encephalopathy, *T2L* T2-hyperintense lesion

^a^*M. tuberculosis*, HIV, hepatitis B and C, virus varicella zoster

^b^Patients without history of chickenpox infection must be vaccinated against varicella zoster virus. Vaccination with live or attenuated vaccines should be completed at least 4–6 weeks before first application of mAb. During treatment, vaccination with live or live-attenuated vaccines is not recommended

^c^Avoidance ingestion of unpasteurized dairy products, raw fish and meat and soft cheeses (2 weeks before, during and at least one month after the infusion). Otherwise, trimethoprim/sulfamethoxazole is recommended for a period of 1 month after the infusion

^d^Complete blood count (hemogram, differential blood count), liver and renal function, thyroid function, urine analysis

^e^Acyclovir 200 mg two times daily $\ge$ 1 month after the last infusion

**Table 2:** Recommendations about family planning in patients with multiple sclerosis receiving mAbs

**DMT**	**Contraception**	**Treatment discontinuation before pregnancy**	**Use in pregnancy**	**Breastfeeding**
Natalizumab	Yes	0–1 month	No^a^	No^b^
Alemtuzumab	Yes	4 months	No	No (≤ 4 months after last infusion)
Rituximab	Yes	6–12 months^c^	No	No (≤ 6 months after last infusion)
Ocrelizumab	Yes	6–12 months^c^	No	No (≤ 6 months after last infusion)
Ofatumumab	Yes	6 months	No	No^d^
Ublituximab	Yes	6–12 months^c^	No	No (≤ 6 months after last infusion)

*DMT* disease-modifying therapy

^a^In case of highly-active disease and upon careful weighing of risk–benefit-ratio and individual discussion with the patient, natalizumab can be used up to 32–34 weeks of gestation

^b^In case of highly active disease and upon careful weighing of risk–benefit-ratio and individual discussion with the patient, natalizumab can be given while breast-feeding

^c^In case of highly active disease and upon careful weighing of risk–benefit-ratio and individual discussion with the patient, contraception may be stopped 3–4 months after the last ocrelizumab/rituximab/ublituximab infusion

^d^In case of highly active disease and upon careful weighing of risk–benefit-ratio and individual discussion with the patient, ofatumumab may be considered during breast-feeding

## Natalizumab

Natalizumab is a humanized second-generation mAb that binds to $\alpha$ 4 integrin receptors on endothelial cells lining blood vessels, disrupting the interaction of $\alpha$4$\beta$1 integrin (or very late antigen 4, VLA-4) expressed on lymphocytes and monocytes with its ligand vascular cell adhesion molecule 1 (VCAM1) on endothelial cells. Thereby, it inhibits migration of leukocytes through the BBB into the brain and spinal cord. While preventing invasion of autoreactive lymphocytes from peripheral blood into the CNS, cells are not depleted from the circulation. Rather, there is an increase in peripheral lymphocyte and monocyte counts during treatment (natalizumab-induced lymphocytosis, NIL) [11, 12]. Natalizumab is approved at a fixed dose of 300 mg administered intravenously or subcutaneously every 4 weeks (standard interval dosing; SID), allowing natalizumab concentrations to be maintained at levels which ensure continuous maximal $\alpha$4$\beta$1 integrin receptor saturation [13].

### Pharmacology and Pharmacodynamics

The median relative bioavailability following intravenous and subcutaneous administration is 100% and 82.4%, respectively [14]. The median half-life of intravenous application of natalizumab is 27.1 days, with the subcutaneous absorption half-life being estimated to be approximately 2.6 days [14]. After absorption, the elimination phase for subcutaneous and intravenous administration parallels each other, suggesting comparable elimination characteristics [14].

Mean natalizumab serum concentrations are lower for extended interval dosing (EID) compared to SID (18.2 vs. 35.7 µg/ml, respectively); besides, $\alpha$_4_-integrin receptor occupancy by natalizumab is lower for EID than SID (78.2% vs. 87.4%, respectively) [15]. As $\alpha$_4_-integrin receptor saturation > 50% correlates with high clinical efficacy of natalizumab, at least 9 natalizumab infusions per year are required to maintain adequate trough saturation and concentrations levels [16]. The pharmacology of natalizumab is mostly affected by body mass index and dosing interval [17].

### Clinical Trials

Both AFFIRM (Natalizumab Safety and Efficacy in RRMS) and SENTINEL (Safety and Efficacy of Natalizumab in Combination with Avonex [IFN $\beta$-1a] in Patients with RRMS) were phase 3 clinical trials assessing the efficacy of natalizumab in RMS [18, 19]. In the AFFIRM study, natalizumab significantly decreased annualized relapse rate (ARR) by 68% (*p* < 0.001) and lowered disability progression rates (sustained for 3 months) by 42% (*p* < 0.001) compared to placebo [18]. Additional analyses showed that over 2 years, natalizumab elicited a 92% and 83% decline in the number of Gd-enhancing lesions and the number of new or enlarging T2L, respectively (both *p* < 0.001). Besides, natalizumab also reduced the rate of brain atrophy during the second year of treatment [18, 20]. The drug is effective in patients with a more severe disease, and has been shown to have beneficial effects on visual function and several aspects of quality of life [21–23].

In the SENTINEL clinical trial, natalizumab plus interferon (IFN) $\beta$-1a significantly reduced the cumulative probability of 12-week confirmed disability progression (CDP) by 24% (*p* = 0.02) and decreased ARR by 55% compared with IFN $\beta$-1a alone (*p* < 0.001) [19].

Long-term data of natalizumab effectiveness from the Austrian MS Treatment Registry show a stable disease course regarding relapse activity and disease progression under natalizumab treatment for more than 7 years, with older age at natalizumab start (> 35 years) being the only significant risk factor for disease progression over long-term [24].

### Safety and Adverse Effects

Although natalizumab is generally well-tolerated and safe, progressive multifocal leukoencephalopathy (PML) may occur as a serious, potentially life-threatening adverse effect.

After natalizumab was first approved in 2004, reported cases of PML led to a withdrawal in 2005 and subsequently reintroduction in 2006 with the establishment of an advisory committee that would monitor patients on natalizumab. PML is an acute or subacutely developing demyelinating disease caused by the John Cunningham virus (JCV) that leads to a lytic destruction of oligodendrocytes. Infection is very frequent but most commonly asymptomatic. It usually occurs during childhood and JCV remains latent until a possible reactivation by mutation of the virus, which remains a very rare event. The presence of anti-JCV antibodies as an indirect footprint of infection, duration of natalizumab exposure (particularly beyond 2 years), and immunosuppressant use prior to receiving natalizumab are all risk factors associated with an increased risk of PML [25]. Anti-JCV antibody-negative patients have an estimated PML risk of 0.07/1000 patients, whereas in anti-JCV antibody-positive patients, estimated cumulative PML probability over 6 years is 2.7% and 1.7% in patients with and without previous immunosuppressive therapy, respectively [26].

### Monitoring and Screening

A comprehensive and exemplary scientific effort by the MS community yielded a clinically applicable risk stratification model. Special anti-JCV antibody index has been developed to predict the risk of PML with people with an antibody index of ≤ 0.9 having an annual PML risk of 0.6/1000, an index of 0.9–1.5 having a risk of 3.0/1000, and an index of > 1.5 having a risk of 10.0 /1000 in 6 years [26]. Re-testing of anti-JCV antibody negative patients every 6 months is recommended [27]. However, patients should not be tested for anti-JCV antibodies within 2 weeks of plasmapheresis given removal of antibodies from the serum, or within 6 months of IVIg [27]. Frequent MRI monitoring (on a 3–6 monthly basis) is recommended for patients who either have all three risk factors (anti-JCV antibody positive, ≥ 2 years of natalizumab treatment, prior immunosuppressant therapy) or patients with a high anti-JCV antibody index who have received at least 2 years of natalizumab treatment without prior immunosuppressant therapy [27]. The extension of the dosing interval from 4 to 6 weeks (EID) has been associated with lower incidence of PML, and no negative effect on efficacy evidenced by ARR, disability progression and MRI activity [28, 29]. Recently published data show that the proportion of relapse-free patients at 72 weeks (97.6% vs. 96.9%), proportion of patients free of disability worsening (92.0% vs. 90.0%), and proportion of patients with No Evidence of Disease Activity (NEDA) (67.4% vs. 70.0%) do not differ between the intervals of application (4 vs. 6 weeks, respectively) [30–32]. Also, real-world evidence indicates equivalent efficacy of SID and EID with EID being safe and well tolerated for over 7 years [33].

Special caution should be exercised when washing out natalizumab in the setting of CNS infection such as PML, since the immune reconstitution inflammatory syndrome (IRIS) in response to viral antigen in the brain may be robust and cause worsening or even death [34].

### Pregnancy and Breastfeeding

Natalizumab is classified as a pregnancy category C drug as potential fetal effects have been reported in animal studies [35–37]. However, there is a risk of reactivation or even rebound of disease activity after natalizumab cessation, which is of particular importance in the first trimester and during the first 3 months postpartum, where disease activity is not yet or not anymore diminished by the effects of pregnancy itself [38–41]. The risk of relapse and disability progression during pregnancy is predicted by pre-conception relapse activity, higher EDSS at conception, use of HET and prolonged washout period [42]. Re-initiating natalizumab administration within 4 weeks after delivery in women without a relapse in the year pre-conception on HET is associated with a ninefold decreased risk for relapse and disability progression postpartum [42]. Thus, there is a clear rationale for continuing natalizumab at least until pregnancy occurs, or in patients with higher disease activity even during pregnancy as antibodies, including natalizumab, only minimally cross the placenta during the first trimester [43–45].

Even though evidence of safety during natalizumab continuation is limited, various expert guidelines incorporated these recommendations. They suggest to continue natalizumab at least until pregnancy is confirmed and, depending on an individual benefit-risk-assessment even until 32–34 weeks of gestation with EID. Natalizumab administration should be resumed as soon as possible after delivery [46, 47].

With reference to the Tysabri Pregnancy Exposure Registry, 355 pregnancy outcomes were analyzed after exposure to natalizumab 3 months before conceiving or during pregnancy. The rate of birth defects and spontaneous abortions was found to be similar to that of the general population [48]. The same findings were obtained in a retrospective analysis from the Austrian MS Treatment Registry [49]. However, in one case series study, mild to moderate thrombocytopenia and anemia were detected in 10 of 13 newborns when natalizumab was prescribed in the third trimester of gestation [50]. It is, therefore, mandatory to test all exposed newborns for thrombocytopenia and anemia [51].

As natalizumab is excreted in breast milk, the SmPC states that breastfeeding should be discontinued during treatment with natalizumab [27]. However, natalizumab concentrations in breast milk are low and large molecules such as natalizumab are most likely destroyed in the infants` gastrointestinal tract. Thus, treatment with natalizumab can be also considered during breast-feeding [47].

### Vaccination

According to EMA, inactivated vaccines can be given to patients receiving natalizumab, whereas live and live-attenuated vaccines have not been studied in those patients and should, therefore, be avoided [27]. There is little evidence on the vaccine response in patients receiving natalizumab. One study confirmed a significant increase in anti-influenza A and B titer after the vaccination in both treated patients and HC, with a lower antibody response to the H3N2 strain [52–54]. Another study demonstrated no difference between immunization response to tetanus toxoid in the presence of natalizumab [55]. Currently available data also indicates comparable humoral immune response to SARS-CoV2 vaccines in patients on natalizumab and healthy controls without the need to discontinue the treatment [56–58]. Therefore, vaccination in patients treated with natalizumab seems to elicit a sufficient immune response.

## Alemtuzumab

Alemtuzumab is a humanized second-generation mAb that binds the CD52 glycoprotein present on lymphocytes, eosinophils, monocytes/macrophages, and dendritic cells but not on hematopoietic progenitors, erythrocytes, or platelets, and elicits rapid depletion of CD52 expressing cells [59, 60]. The function of CD52 is not well understood, but evidence suggests that it may be involved in T cell co-stimulation and migration [61]. The dosing consists of 5 consecutive days of infusions at treatment initiation followed by 3 consecutive days of infusions 12 months later, with optional additional courses per approved local labels [62].

Alemtuzumab was the first monoclonal antibody used for therapeutic purposes. Originally, FDA approved it in 2001 for use in B-cell chronic lymphocytic leukemia. It became FDA-approved for use in RMS in 2014. However, because of the risk of autoimmune disorders and due to rare but severe vascular effects, its use has been recommended to be restricted to patients who have failed at least two other DMT approved for RMS.

### Pharmacology and Pharmacodynamics

Following cell surface binding of alemtuzumab to lymphocytes, alemtuzumab results in the depletion of circulating CD52-positive cells in a rapid manner, and the proposed mechanism of lymphocyte depletion includes both antibody-dependent cell-mediated cytolysis (ADCC) and complement-dependent cytolysis (CDC) [60, 63, 64]. As alemtuzumab is administered intravenously, its bioavailability is 100%. It does not cross cell membranes and is expected to distribute between the plasma and interstitial space. Its half-life is approximately 4–5 days and low or undetectable serum concentrations were measured within 30 days after last infusion [62].

Alemtuzumab induces a prolonged lymphopenia, with B-cell counts returning to the lower limits of normal ($\ge$ 0.1 $\bullet$ 10^9^/l) within 7 months, CD8 + cell counts ($\ge$ 0.2 $\bullet$ 10^9^/l) within 20 months, and CD4 + cell counts ($\ge$ 0.4 $\bullet$ 10^9^/l) within 35 months; however, T-cell counts rarely recover to their pretreatment levels [65, 66]. A hyper-repopulation of immature B cell clones to 160–180% of baseline levels is observed at 3–6 months [67]. The peculiar reconstitution of the B-cell compartment has been suggested to be at the root of the development of secondary autoimmunity that is frequently observed in alemtuzumab-treated patients.

However, lymphopenia in absolute number does not seem to be the driving force behind alemtuzumab’s efficacy and safety profile; besides, the rate of lymphocyte count reconstitution seems to be unrelated to relapse risk, infection, or secondary autoimmunity [68, 69]. Moreover, the distinctive pattern of repopulation that begins within weeks and continues over time indicates a possible rebalancing of the immune system, which persists beyond the actual course of treatment. Alemtuzumab treatment results in a relative increase of cells with memory and regulatory phenotypes and a decrease in cells with a proinflammatory signature, and therefore, further promotes an immunoregulatory environment through an impact on other innate immune cells (e.g., dendritic cells) that play a role in MS pathogenesis [70, 71].

### Clinical Trials

The efficacy and safety of alemtuzumab compared to that of IFN $\beta$-1a was shown in two phase 3 randomized, controlled, clinical trials called CARE-MS I and CARE-MS II. CARE-MS I enrolled only treatment-naïve patients [72, 73]. Alemtuzumab significantly decreased ARR (49.4–54.9%): It was associated with a significant reduction in 6-month CDP in CARE-MS II but not in CARE-MS I [72, 73]. Alemtuzumab was superior to IFN $\beta$-1a in reducing the number of Gd-enhancing lesions (9% vs. 23% at year 2, respectively) and new or enlarging T2L (46% vs. 68%, respectively) in both studies [74]. Besides, there were higher proportions of patients free from disease activity during the second year of therapy in the alemtuzumab-treated group in both studies (50% vs. 30–40%). Alemtuzumab also diminished the extent of brain atrophy over 2 years by 40% and 25% in CARE-MS I and CARE-MS II, respectively (*p* < 0.001 and *p* = 0.012).

Furthermore, durable efficacy was demonstrated throughout the extension studies, with 62% of patients having NEDA, and the majority of patients (50–68.5%) not requiring retreatment with alemtuzumab or another DMT for 9 years [75–78]. Imaging data of alemtuzumab-treated patients in exploratory studies have demonstrated potential neuroprotective effects, with increased retinal nerve fiber layer thickness consistent with reduced neurodegeneration, increased myelin water fraction suggestive of remyelination, and stabilized magnetization transfer ratio indicating preserved myelination [79–82].

### Safety and Adverse Effects

In the clinical trials, several adverse effects were reported, with infusion-associated reactions being the most common, occurring in more than 90% of participants [83]. Infusion-associated reaction comprises symptoms like headache, rash, fever, nausea, vomiting, and myalgia, which are part of the so-called cytokine release syndrome and decrease in their occurrence and severity over the course of repeated infusion [84]. They occur within 2–6 h after alemtuzumab infusion. The introduction of high dose methylprednisolone intravenously before alemtuzumab infusion has dramatically reduced infusion-associated reactions [85].

Among side effects, infections were mostly mild or moderate due to the preservation of the innate immune system, with a peak after the first course (66–77%) and declining over time [86–88]. The most common infections reported in Clinical trials were upper and lower respiratory tract infections (nasopharyngitis, sinusitis, flu, bronchitis, pneumonia), masticatory and digestive tract infections (oral herpes, dental infections, gastroenteritis, appendicitis), infection of the urinary tract, and superficial fungal infections (oral and vaginal candidiasis) [89].

A rare but serious infection that has been associated with alemtuzumab is listeriosis, an infection with Gram-positive bacteria *Listeria monocytogenes*, which is usually contracted from unpasteurized dairy products, raw fish and meat, and soft cheeses. Immunocompetent persons rarely develop severe symptoms, whereas defective cellular immunity or pregnancy increase the risk of developing septicemia, meningitis or encephalitis with a mortality rate 20–40% [90, 91]. Furthermore, alemtuzumab administration has been associated with higher rates of HSV infections, sometimes even requiring hospitalization, and VZV infections [72, 73]. Therefore, FDA-approved product label recommends prophylaxis with acyclovir from the start of treatment until CD4 + lymphocytes recover to at least 200 cells/µl, with a minimum duration of prophylaxis of 2 months even if CD4 + lymphopenia resolves earlies [62]. In order to reduce the risk of *L. monocytogenes* infection, patients are advised to keep a Listeria-free diet at least 2 weeks before, during, and 1 month after each infusion [62]. If prophylactic measures are insufficient or unattainable, antibiotic prophylaxis with trimethoprim/sulfamethoxazole should be considered for the period of 1 month after the last infusion. Although serious opportunistic infections have been observed, they occur very rarely [92].

Development of autoimmune diseases is probably the most relevant risk of the treatment with alemtuzumab. Although it remains unclear why only a subset of patients develops autoimmune side effects, hyperrepopulation of B lymphocytes is likely to be a major driver [93]. Elevated levels of interleukin (IL) 21 have been suggested to be predictive of this secondary autoimmunity but this remains contentious [94, 95]. Secondary autoimmune disorders can occur up to 5 years after treatment with a frequency peak at 12–18 months [93]. The most commonly reported autoimmune adverse effect is thyroid dysfunction with either hyper- or hypothyroidism, reported in approximately 36% of patients in a 4-year follow-up of the CARE-MS I and CARE-MS II trials [96]. In the case of hypothyroidism, thyroid hormone replacement therapy should be considered, with patients monitored every 4–8 weeks to adjust thyroid hormone dosages. Hyperthyroidism following alemtuzumab treatment is most likely due to Graves’ disease and should be managed initially with anti-thyroid medication which has been associated with a high likelihood of remission. Thyroidectomy or radioactive iodine would only be indicated following failure of anti-thyroid medication. Where subacute painless thyroiditis is suspected, β-adrenergic blockers or corticosteroids in severe cases may be considered, but not anti-thyroid medications as thyroid hormone synthesis in those patients is already low [96, 97].

Immune thrombocytopenic purpura (ITP) is also one of the potential autoimmune conditions and has been detected in approximately 2% of patients. It is in most instances responsive to first-line therapy with corticosteroids, platelet replacement, and/or intravenous immunoglobulins [98, 99]. Apparently, the risk of this complication is not further increased in the subset of patients receiving additional alemtuzumab beyond the initial two courses [100]. Rarely, nephropathies such as Goodpasture disease with anti-glomerular basement membrane (anti-GBM) antibodies also occur [101].

In a recently published study, five patients received at least one infusion of low-dose rituximab following alemtuzumab treatment, with none of them developing secondary autoimmune disorders [98]. This speaks in favor of the imbalance in B- and T-cell regulatory networks during immune reconstitution as the driving force of autoimmune disorders following alemtuzumab treatment.

In the postmarketing surveillance phase, additional serious safety concerns of cardiovascular complications were identified [102, 103]. Among those, cardiac ischemia and myocardial infarction (2.0/10,000), ischemic and hemorrhagic stroke (3.6/10,000), arterial dissection (1.6/10,000), pulmonary hemorrhage and embolism (4.3/10,000), and vasculitis seem to be those of greatest concern [62, 92, 104]. The underlying pathophysiology remains to be elucidated. Cytokine-release syndrome caused by increased levels of serum tumor necrosis factor (TNF), IFN, and IL-6, leading to vasospasm or transient myocardial dysfunction have been pathomechanistically invoked [105, 106]. Another potential explanation could be direct cardiac myocytotoxicity causing myocyte dysfunction or electrical disturbances [107–109].

Beyond well-known adverse effects, rarer but still significant serious adverse events have been reported in patients during and following alemtuzumab treatment, e.g., exacerbated CNS inflammation with tumefactive demyelination, acute cholecystitis, vasculitis, sarcoidosis, listeria meningitis and meningoencephalitis, hemolytic anemia, hemophagocytic lymphohistiocytosis, opportunistic infections, and acute pneumonitis and pericarditis [110–122].

Several cases of malignancy have also been reported in patients receiving alemtuzumab, but causality is not established. It may represent a random finding because of effective monitoring bias [123]. Reported malignancies encompass papillary thyroid cancer, basalioma, non-EBV-associated Burkitt’s lymphoma, breast cancer, and cancer of the uterus.

### Monitoring and Screening

On the basis of reported side effects recommendations have been formulated. Baseline routine screening of blood (thyroid panel, cell count inclusive of CD4/CD8 ratio, liver function tests, basic metabolic panel, HIV, HBV, HCV, VZV, and $\beta$-HCG), dermatologic examination and urinalysis within 30 days prior to the first infusion should be conducted. Thereafter, cell counts (inclusive of CD4/CD8 ratio), TSH, creatinine should be determined and urinalysis performed every month, and dermatologic examination performed yearly for 5 years after the last treatment cycle.

Prophylaxis with oral antiviral (acyclovir) is commenced one week prior to the first infusion and discontinued when CD4 count $\ge$ 200, and listeria prophylaxis with listeria-free diet or co-trimoxazole is recommended. Patients are also pretreated with steroids, antihistamines and acetaminophen on infusion day.

### Pregnancy and Breastfeeding

Alemtuzumab is classified as a pregnancy category C drug, as alemtuzumab was embryolethal in pregnant huCD52 transgenic mice when administered during organogenesis [62]. According to the Summary of Product Characteristics (SmPC), serum concentrations of alemtuzumab are low or undetectable within 30 days of each treatment course [62]. Therefore, women of childbearing potential should use effective contraception when receiving a course of alemtuzumab, and for 4 months following each course of treatment [124]. A study analyzing pregnancy outcomes in women treated with alemtuzumab, reported 66% healthy live births, 22% spontaneous abortions, 11% elective abortions, and 0.6% stillbirth (*n* = 167) [125]. Maternal age seemed to be associated with an elevated risk of spontaneous abortion (relative risk [RR] 2.46 in patients $\ge$ 35 years) [126]. However, the risk of spontaneous abortion was not increased in patients becoming pregnant $\le$ 4 months versus > 4 months since alemtuzumab exposure (19% vs. 23%, RR 1.08) [126]. The risk of autoimmune thyroid disease remains increased for 4 years after completing alemtuzumab treatment, therefore thyroid function should be tested regularly in newborns [124, 127].

Although it is unclear whether alemtuzumab is excreted in human breast milk, it falls into class C category as it has been detected in the milk of lactating mice. Hence, women should be advised to discontinue breastfeeding during each course of treatment, and for at least 4 months after each course [128].

As a cyclically administered treatment, alemtuzumab may be considered in women with very high disease activity and without acute plans to become pregnant.

### Vaccination

According to EMA and FDA label, inactivated vaccines can be given to patients receiving alemtuzumab, whereas live and live-attenuated vaccines have not been studied in those patients and should, therefore, be avoided [128]. The SmPC suggests that vaccination before alemtuzumab should be considered in patients who have not completed standard required vaccinations, and for those without immunity to chickenpox [62]. Required vaccinations should be given at least 6 weeks before treatment [62].

The ability to mount effective immune responses to vaccines following alemtuzumab has not been studied extensively. Diphtheria, tetanus, poliomyelitis, and pneumococcus vaccines have been shown to evoke a normal T-cell response upon administration in patients treated with alemtuzumab despite the relatively prolonged T- and B-cell suppression [129, 130]. However, one patient was vaccinated within two months of alemtuzumab treatment and developed a poor response to several vaccines, suggesting immunization very early after alemtuzumab may not be effective [129]. Currently available data also indicates nearly normal humoral immune response to SARS-CoV2 vaccines in patients on alemtuzumab and healthy controls, depending on lymphocyte counts and time since last application of alemtuzumab [56, 58, 131].

## B-Cell Depletion Therapy

mAbs targeting CD20-expressing lymphocytes B represent an important treatment option for patients with MS. Spared from anti-CD20 lysis are stem cells (pro-B cells), many plasmablasts, and terminally differentiated antibody-producing plasma cells [132]. Anti-CD20 mAb further differ in their structure (chimeric, humanized, fully human), relative potency to drive ADCC and CDC, route of administration (intravenous or subcutaneous), pharmacokinetics, and required infusion times (Table 3) [133]. Three anti-CD20 mAbs are currently available with ocrelizumab and ofatumumab labeled for treatment of MS and rituximab frequently used off-label. Another one, ublituximab, is expected to be approved in 2022.

**Table 3:** Overview of anti-CD20 mAb for the treatment of MS

	Rituximab	Ocrelizumab	Ofatumumab	Ublituximab
				
Molecular structure	Chimeric murine/human IgG1 kappa	Recombinant humanized glycosylated IgG1	Fully human IgG1 kappa	Chimeric IgG1 with glycoengineered Fc segment
Human sequence	65%	> 90%	100%	65%
Molecular weight	~ 145 kDa	~ 145 kDa	~ 146 kDa	~ 144.5 kDa
Immunogenicity	+ + +	+ +	+	+ +
Mechanism of B-cell depletion				
ADCC	+ +	+ + +	+ +	+ + + +
CDC	+ +	+	+ + +	+

*ADCC* antibody-dependent cell cytotoxicity, *CDC* complement-dependent cytotoxicity, *IgG* immunoglobulin G

### Rituximab

Rituximab is a second-generation chimeric mouse-human anti-CD20 mAb that was approved in 1997 for B-cell lymphoma but is being used off-label in several neurological diseases, including neuromyelitis optica spectrum disorder (NMOSD), myasthenia gravis, and MS. Several different protocols of rituximab dosage have been used, with patients being most commonly treated with 500 or 1000 mg rituximab intravenously every 6–12 months, in some cases after two initial application held 2 weeks apart [134–136].

#### Pharmacology and Pharmacodynamics

Rituximab works primarily through CDC of B cells but also has significant ADCC activity. Due to its intravenous route of application, its bioavailability is 100%. The replenishment of B cells is subject to individual variability, with a study with 26 RRMS patients showing a reconstitution to a mean of 35% of baseline counts by week 72, with the vast majority being naïve B cells rather than memory B cells [137]. The elimination half-life for intravenous rituximab 2 times 1000 mg administered 2 weeks apart is around 20 days but depends on sex, body weight, and renal clearance [138].

#### Clinical Trials

In spite of the overall positive efficacy with only rare serious adverse events, rituximab was never tested in phase 3 trials for efficacy in RMS. However, there is growing evidence from real-world evidence studies strengthening the case for rituximab as a potent treatment option for RMS [135, 139, 140].

IRRs are relatively common with use of rituximab in MS, appearing in 67.1–78.3% of treated patients after first infusion compared to 23.1–40.0% placebo-treated patients [141, 142]. However, they decrease to placebo levels with successive infusions, are only mild to moderate in severity, and include fever, rush, chills, throat irritation, nausea, headache, cough, tiredness, headache, hypotension, bronchospasm, or angioedema [141, 143].

Besides, treatment with rituximab is associated with an increased risk of infections [144]. Serious infections occur in 4.5% of treated patients compared to < 1.0% in placebo-treated patients with no clear association to the number of infusions [141]. Patients treated with rituximab should be screened for hypogammaglobulinemia and neutropenia, as these may present independent risk factors for developing infections [145, 146].

There is an increased PML risk with rituximab treatment (adjusted odds ratio = 3.22), but lower when compared to that of natalizumab [147]. Beside PML, reactivation of other latent infections such as tuberculosis, hepatitis, or HIV upon rituximab treatment has been reported [148, 149]. Therefore, patients should be thoroughly screened for such infections prior to rituximab treatment.

A low frequency of all types of malignancies was reported for rituximab in MS patients, which did not differ significantly from the general population (26.6 vs. 28.9 per 10,000 patient years, respectively) [150].

#### Monitoring and Screening

Cases of hepatitis B reactivation have been reported in subjects receiving anti-CD20 mAb [151]; therefore, HBV screening should be performed in all patients before initiation of treatment (HBsAg, HBcAg) [152]. Patients with active hepatitis B disease should not be treated with rituximab [153].

Apart from routine laboratory tests, baseline immunoglobulin levels should be determined as a reduced baseline level of IgG has been associated with higher risk for severe infections with rituximab in patients with rheumatoid arthritis [144]. Currently, there is no evidence to suggest monitoring anti-JCV antibodies in patients on rituximab.

#### Pregnancy and Breastfeeding

Rituximab is classified as a pregnancy category C drug as there are no adequate and well-controlled studies of rituximab in pregnant women [153]. Although at least a period of 6–12 months (FDA/EMA) after the last injection of rituximab is recommended before conceiving, a study analyzing 90 live birth outcomes of women inadvertently conceiving during or less than 12 months after the treatment of rituximab reported 22 premature births, one neonatal death after 6 weeks, 11 newborns with hematological changes (B-cell deficiency, neutropenia, thrombocytopenia, anemia, and lymphopenia), and two inborn malformations [154]. Besides, a recent systematic review and case series of MS and NMOSD patients assessed the safety of rituximab before and during pregnancy, with no major safety signal being found with rituximab use withing 6 months of conception [155]. However, as anti-CD20 mAbs can be actively transported across placental barrier and subsequently deplete fetal B cells, women are advised to use effective contraception for at least 3–4 months after the last rituximab infusion [47, 155, 156].

A case report from a breastfeeding patient found 0.42% of rituximab serum concentration in the milk, and similar concentrations were found in monkeys as well (0.19–0.26%) [157, 158]. As IgG is degraded in the gut of newborns, administration of rituximab is highly unlikely to pose clinically relevant risk for the infant, but any recommendation regarding its use in breastfeeding women should await more safety data [157]. To avoid potential harm to the newborn, women are still advised not to breastfeed during and up to 6 months after discontinuing the treatment.

#### Vaccination

EMA and FDA labels allow inactivated vaccines to be given to patients receiving rituximab, whereas live and live-attenuated vaccines have not been studied in those patients and should, therefore, be avoided [153].

Response to vaccination in patients receiving rituximab was only studied in non-MS populations. Patients with rheumatoid arthritis showed a reduced response to pneumococcal vaccine when treated with both rituximab and methotrexate compared to methotrexate alone (57% vs. 82%, respectively) [159].

Available data indicates significantly reduced humoral immune response to SARS-CoV2 vaccines (15–60% developing antibodies) in patients on rituximab compared to healthy controls, depending on B cell counts and time since last application [56, 58, 160]. Moreover, the development of a humoral immune response remains rare in seronegative patients with MS on anti-CD20 mAb even after a third dose of SARS-CoV-2 vaccine unless patients have measurable B-cell counts [161]. However, there is growing evidence that T cell responses may be preserved or even augmented under anti-CD20 mAbs, potentially mitigating the loss of antibody-mediated vaccine efficacy [162, 163].

Therefore, every patient considered for rituximab therapy should receive all indicated vaccines (hepatitis B for at-risk population, pneumococcus, tetanus toxoid every 10 years, influenza annually) before treatment. Ideally, vaccination should be undertaken at least 4 weeks before treatment initiation [164].

### Ocrelizumab

Ocrelizumab is a second-generation recombinant humanized mAb targeting CD20-expressing B cells that is approved for RMS and primary progressive MS [165]. According to the label, it is administered with two starting doses of 300 mg 2 weeks apart, and after that 600 mg every 6 months. Patients should be premedicated at least 30–60 min prior ocrelizumab infusion with 100 mg methylprednisolone and an antihistamine in order to avoid IRRs. Patients need to be observed for at least 60 min following ocrelizumab infusion [166].

#### Pharmacology and Pharmacokinetics

Ocrelizumab binds to the extracellular loop of CD20 causing ADCC and CDC of circulating B cells [143, 166]. Interestingly, recent studies have shown that ocrelizumab may also target CD20 + T cells, which are present in low frequencies in MS patients, suggesting an alternative contributing mechanism of action [167]. As it is administered intravenously, its bioavailability is 100%. The median time to B-cell repletion was 72 weeks, with 90% of patients reaching pre-treatment levels by approximately 2.5 years after the last infusion [168]. Ocrelizumab has a half-life of 26 days [165]. It is expected to enter the metabolic pathway of endogenous antibodies; in that way, no studies concerning its metabolism and elimination were performed [168].

#### Clinical Trials

The efficacy and safety of ocrelizumab versus IFN $\beta$-1a for the treatment of RMS was reported in two phase 3 clinical trials named OPERA I and OPERA II [169]. In both trials, treatment with ocrelizumab lowered ARR (0.16 vs. 0.29; *p* < 0.001), and led to lower percentage of patients with CDP at 12 weeks (9.1% vs. 13.6%, *p* < 0.001) and lower number of Gd-enhancing lesions. 47.9% and 47.5% of ocrelizumab-treated patients (OPERA I and II, respectively) had no evidence of disease activity at 96 weeks compared to 29.2% and 25.1% on IFN $\beta$-1a, respectively [170].

#### Safety and Adverse Effects

IRRs occurred in 34.3% of the treated patients with ocrelizumab (vs. 9.7% on IFN $\beta$-1a); a shorter infusion period (2 h instead of 3.5 h) did not increase the risk of IRRs in one recently published study [169, 171]. Although CDC activity was believed to play an important role in triggering infusion-related reactions, IRRs seem to be mainly associated with cytokine release by immune cells (lymphocytes B and natural killers) [172, 173]. Current recommendations to reduce the risk of an IRR include premedication with methylprednisolone and an antihistamine [171].

The most common adverse events are infections with the overall rate of 84.5% in the period up to 8 years [174–176]. Most common infections were upper respiratory tract infections (predominantly nasopharyngitis) and urinary tract infections. Serious infections occurred in 1.3% of patients treated with ocrelizumab (vs. 2.9% on IFN $\beta$-1a) [169]. Approximately 30% of patients show hypogammaglobulinemia, which significantly increases infection risk.

Neoplasms occurred in 1.1% of patients treated with ocrelizumab and 0.4% patients treated with IFN $\beta$-1a [169, 174]. From those, 6 were breast cancer cases, while no such cases were observed in the placebo or IFN $\beta$-1a group. The total number of patients with breast or other cancers in the ocrelizumab-treated populations was, however, not higher than expected as background from epidemiological studies of the general population. Also, the incidence of cancer has fallen during the subsequent open-label extension studies [175, 177].

Currently, 8 cases of PML have been identified in patients treated with ocrelizumab, which were judged related to previous treatment with natalizumab or fingolimod, while one PML case was considered to be directly associated with ocrelizumab treatment as the patient had no prior DMT exposure (progressive MS) [175, 178].

#### Monitoring and Screening

Cases of hepatitis B reactivation have been reported in subjects receiving anti-CD20 mAb; therefore, HBV screening should be performed in all patients before initiation of treatment (HBsAg, HBcAg) [151]. Patients with active hepatitis B disease should not be treated with ocrelizumab [168]. Cases of late-onset neutropenia have been reported, with the majority being reported at least 4 weeks after last ocrelizumab infusion (grade 1 or 2). In patients with signs and symptoms of infection, measurement of blood neutrophils is recommended.

Given the observation of malignancies in the pivotal trials, patients with a known active malignancy should not be treated with ocrelizumab, and every patient should follow standard breast cancer screening per local guidelines [168]. There is no evidence to support monitoring anti-JCV antibodies in patients treated with ocrelizumab.

#### Pregnancy and Breastfeeding

Ocrelizumab is classified as a pregnancy category C drug as there are no adequate data on the developmental risk associated with the use of ocrelizumab in pregnant women [168]. According to EMA, animal studies do not indicate teratogenic effects of ocrelizumab, but B cell depletion was detected in utero and reproductive toxicity was observed in pre- and post-natal development studies [168]. Besides, ocrelizumab is a humanized mAb of an immunoglobulin G1 subtype that is known to cross the placental barrier. Therefore, women of childbearing potential are advised to use contraception while receiving ocrelizumab and for 12 months after the last infusion [168].

As this label appears very conservative given the available pharmacological data, a growing number of experts and guidelines recommends women to use effective contraception for at least 3–4 months after the last ocrelizumab infusion [47, 155].

Recently, a study of the German MS and Pregnancy Registry was published claiming B cells to be normal in infants breastfed by mothers receiving anti-CD20 mAb [179]. However, women are still advised to discontinue breastfeeding during ocrelizumab therapy [168].

#### Vaccination

Current EMA and FDA labelling allows application of inactivated vaccines to patients receiving ocrelizumab. Live or live-attenuated vaccines have not been studied in those patients and should, therefore, be avoided during treatment and until B-cell repletion [168]. The VELOCE study, which evaluated the effects of ocrelizumab on immune response to various vaccines in patients with RMS, confirmed that patients treated with OCR can mount humoral responses, albeit attenuated, to the inactivated vaccine studied (tetanus, pneumococcal and influenza vaccine) [180]. Available data indicates significantly reduced humoral immune response to SARS-CoV2 vaccines in patients on ocrelizumab compared to healthy controls, depending on B cell counts and time since last application [56, 58, 160, 164, 181, 182]. Therefore, some authors suggest extending dosage intervals in order to improve chances of building up a sufficient immune response [183]. However, it has to be kept in mind that many patients (37–53%) still develop humoral response under ocrelizumab and that T cell response seems to be unaffected under B cell depletion [58, 184–186].

Patients should be reviewed for their immunization status before embarking on treatment with ocrelizumab. Patients who require vaccination should complete it at least 6 weeks prior to treatment initiation. It is recommended to vaccinate patients with ocrelizumab with seasonal influenza vaccines that are inactivated [168].

### Ofatumumab

Ofatumumab is a fully human mAb targeting CD20-positive B cell lineage cells but recognizing a different epitope than either rituximab or ocrelizumab. It was originally approved by the FDA in 2009 for use in chronic lymphocytic leukemia but has been also approved for use in MS in 2020.

#### Dosage and Administration

Its notable strength is its subcutaneous application with an auto-injector pen which is administered at four-week intervals with the first three doses delivered on days 1, 8, and 15. Despite its differing route of application, it does not seem to be inferior to other mAbs used in treatment of MS [187].

#### Pharmacology and Pharmacokinetics

Ofatumumab binds to an epitope encompassing both small and large loops of the extracellular domain of the CD20 protein, causing ADCC and CDC of circulating B cells [188]. The mechanisms are similar to ocrelizumab, although ofatumumab causes more CDC than ADCC, and in this regard, resembles rituximab [189]. Its bioavailability is 85% and 40% on day 1 and day 15, respectively [190]. After several subcutaneous applications of ofatumumab, its half-life is 16 days.

Low-dose subcutaneous ofatumumab treatment provides effective B cell depletion within lymphoid tissues, comparable to high-dose intravenous rituximab. However, subcutaneous administration may facilitate ofatumumab entry into lymphatic drainage and lymph nodes [191]. Before reaching the maintenance dose by week 4, 94% of patients had levels of B lymphocytes < 10 cells/µl. Pre-depletion levels of B cells are reached in 24.6 weeks after treatment discontinuation [190]. Modes of metabolism and excretion are anticipated to be similar to endogenous antibodies, but no studies were performed specifically with ofatumumab.

#### Clinical Trials

The efficacy and safety of ofatumumab was investigated in two double-blind, double-dummy phase 3 clinical trials called ASCLEPIOS I and ASCLEPIOS II with teriflunomide as an active comparator [192]. ARR was lower with ofatumumab in both studies (0.11 vs. 0.22 ASCLEPIOS I; 0.10 vs. 0.25 in ASCLEPIOS II). The decrease in the number of Gd-enhancing lesions was greater with ofatumumab (0.01 vs. 0.45 in ASCLEPIOS I; 0.03 vs. 0.51 in ASCLEPIOS II) and the numbers of new or enlarging lesions per year (0.72 vs. 4.00 in ASCLEPIOS I; 0.64 vs. 4.15 in ASCLEPIOS II) were lower than with teriflunomide. In the pooled trials, the percentage of patients with CDP at 3 and 6 months was 10.9% and 8.1% with ofatumumab and 15.0% and 12.0% with teriflunomide, respectively (hazard ratio 0.66 and 0.68, respectively). The rate of brain atrophy did not differ significantly between the ofatumumab group and the teriflunomide group (− 0.28% and − 0.29% with ofatumumab and -0.35% with teriflunomide in ASCLEPIOS I and ASCLEPIOS II, respectively) [192].

#### Safety and Adverse Effects

The most common adverse effect was the injection-related reaction which occurred in 20.6% in the ofatumumab group but also in 15.0% in the teriflunomide group [192]. The injection-related reaction is most marked after the first application (14.4%) and seems to diminish subsequently (4.4% after the second, < 3% after the third application) [192]. The most commonly reported symptoms were fever, headache, myalgia, and fatigue.

Serious infections occurred in 2.5% and 1.8% of the patients in the respective groups [192]. Most common were upper-respiratory (39.4%) and urinary tract infections (11.9%) which were mostly mild to moderate. Like other B-cell depleting therapies, ofatumumab causes hypogammaglobulinemia, although there is currently no evidence indicating an elevated risk for infections in patients these patients.

Five neoplasms (0.5%) occurred in the ofatumumab group (two cases of basal-cell carcinoma and one case of malignant melanoma, recurrent non-Hodgkin’s lymphoma, and invasive breast carcinoma, each) and four (0.4%) in the teriflunomide group.

#### Monitoring and Screening

Since hepatitis B reactivation can occur in patients treated with anti-CD20 mAb; patients with active hepatitis B disease should not receive ofatumumab, and HBV screening should be performed in all patients before initiation of treatment (HBsAg and HBcAb) [151, 190].

#### Pregnancy and Breastfeeding

Ofatumumab is classified as a pregnancy category C drug as there are no adequate or well-controlled studies of ofatumumab in pregnant women [190]. Recently, a study on cynomolgus monkeys proved that intravenous application of ofatumumab from gestation day 20 until parturition does not affect pre- or postnatal development [193]. As of 31 August 2021, 32 pregnancies were reported in women with MS exposed to ofatumumab; no birth defects or congenital anomalies were reported in 23 pregnant women with known outcomes [194]. However, as ofatumumab crosses the placental barrier and fetuses exhibit depletion of peripheral B cells and decreased spleen and placental weights, treatment with ofatumumab should be avoided during pregnancy unless the potential benefit to the mother outweighs the potential risk to the fetus [190, 194].

No information is available on the clinical use of ofatumumab during breastfeeding. However, as ofatumumab is a large protein molecule (146 kDa), its amount in milk is likely to be very low, confirmed by some studies evaluating transfer of other mAb into breastmilk with comparable molecular weight [195, 196]. Furthermore, it is also partially destroyed in the infant’s gastrointestinal tract and absorption by the infant will be minimal. Therefore, if clinically needed, ofatumumab can be used during breast-feeding [190].

#### Vaccination

According to EMA and FDA, inactivated vaccines can be administered to patients receiving ofatumumab, whereas live or live-attenuated vaccines have not been studied in these patients and should, therefore, be avoided during treatment and after discontinuation until B cell repletion [190]. Immunization with live or live-attenuated vaccines should be performed at least 4 weeks prior to initiation of ofatumumab whereas at least 2 weeks should elapse before immunization with inactivated vaccines [190].

The safety of and ability to generate an antibody response to vaccination during treatment with ofatumumab has not been studied yet. The response to vaccination could be, however, impaired when lymphocytes B are depleted, which of course also applies to SARS-CoV2 vaccination [58, 160, 190].

### Ublituximab

Ublituximab is a novel chimeric mAb against CD20-positive lymphocytes B that targets an epitope on CD20 not targeted by other anti-CD20 mAb, allowing lower doses and shorter infusion times in comparison to other anti-CD20 mAb [189, 197]. It has been glycoengineered to exhibit a low-fucose fragment crystallizable (Fc) region, demonstrating 100 times greater ADCC in vitro than rituximab in cells from patients with chronic lymphocytic leukemia [197, 198]. This activity is evident regardless of CD20 surface expression level on target cells as opposed to ofatumumab which demonstrates superiority to CDC-mediated killing of target cells expressing high levels of CD20 only [199]. All patients reached $\ge$ 95% B cell depletion from baseline within 2 weeks after the second ublituximab infusion, with depletion occurring already within 24 h of the initial dose in most patients. B cell depletion was sustained pre-dose at weeks 24 and 48 [200].

The efficacy and safety of ublituximab was investigated in ULTIMATE I and ULTIMATE II clinical trials using teriflunomide as an active comparator, with patients being randomized to receive ublituximab 150 mg on day 1, and 450 mg on day 15, and weeks 24, 48, and 72. Their primary endpoint, ARR after 96 weeks of treatment, was reduced in both studies (0.08 vs. 0.19 [59.6%] and 0.09 vs. 0.18 [48.9%], respectively). A pooled analysis of CDP from both ULTIMATE studies at 12 and 24 weeks showed a 15.7% and 34.3% reduction for ublituximab compared to teriflunomide, although this was not statistically significant. There was a strong reduction of the total number of Gd-enhancing lesions (lesions per scan per participant: 0.02 vs. 0.49 [96.7%] and 0.01 vs. 0.25 [96.4%], respectively), and the number of new or enlarging T2L (0.21 vs. 2.79 [92.4%] and 0.28 vs. 2.83 [90.0%], respectively), while post hoc analysis of brain volume change between week 24 and 96 showed no difference between treatment arms. NEDA was reached in 43.0–44.6% of patients on ublituximab, and in 11.4–15.0% on teriflunomide (*p* < 0.0001) [201]. Ublituximab also demonstrated significant improvement in the overall MSFC scores in both ULTIMATE I and II (*p* = 0.0484 and *p* = 0.0171, respectively), with 9HPT being statistically significant in both groups and T25FW in ULTIMATE II but not in ULTIMATE I [202].

A single-arm extension study of those studies was initiated in November 2019 to study the long-term efficiency and safety profile of ublituximab; results from the open-label extension are expected in 2023.

Ublituximab was generally well tolerated, and the most common adverse effect was an IRR, occurring in 43.4% of patients (most commonly grade 1 or 2) [200, 201]. These were most frequent at the first dose, and decreased in frequency with subsequent dosing [201]. Respiratory tract infections occurred in 15.0–17.2% of patients [200, 201]. Proportion of patients with IgM levels under the lower limit of normal after week 96 was 20.9% in the ublituximab and 4.9% in the teriflunomide group [201].

Serious adverse events were reported in 52 (9.5%) patients, the most common being infections (4.0%) and nervous system disorders (0.9%). In total, two malignancies were reported (endometrial and uterine cancer). Three deaths occurred in patients treated with ublituximab due to encephalitis (post-measles), salpingitis and pneumonia, the latter being possibly related to treatment [201]. No case of PML was reported.

Currently, no data are available for the use of ublituximab during pregnancy and breast feeding nor is there any published data available on vaccination. As a member of the anti-CD20 mAb class, recommendations are based on other anti-CD20 mAb, including SARS-CoV2 vaccination.

## Conclusion

mAb have become a mainstay of treatment in patients with MS who are in need of HET. The arsenal will most likely be further broadened by the approval of ublituximab in 2022. Further investigations will analyze safety and efficacy of different administration regimes. While all mAb in use have shown high efficacy, serious adverse events may occur with different frequency and require appropriate monitoring and risk management.

## Supplementary Information

Below is the link to the electronic supplementary material.

Supplementary file1 (PDF 2826 KB)

Supplementary file2 (PDF 2386 KB)

Supplementary file3 (PDF 2378 KB)

Supplementary file4 (PDF 2984 KB)

Supplementary file5 (PDF 2321 KB)
